# Development of novel long noncoding RNA MALAT1 near-infrared optical probes for *in vivo* tumour imaging

**DOI:** 10.18632/oncotarget.20652

**Published:** 2017-09-05

**Authors:** Meng-Jie Dong, Cai-Qin Wang, Guo-Lin Wang, Yue-Hong Wang, Zhen-Feng Liu

**Affiliations:** ^1^ The Department of Nuclear Medicine, The First Affiliated Hospital, College of Medicine, Zhejiang University, Hangzhou 310003, China; ^2^ Key Laboratory of Precision Diagnosis and Treatment for Hepatobiliary and Pancreatic Tumor of Zhejiang Province, Hangzhou 310003, China; ^3^ Key Laboratory of Reproductive Genetics (Zhejiang University), Ministry of Education, Zhejiang Province, 310003, China; ^4^ Department of Respiratory Medicine, The First Affiliated Hospital, College of Medicine, Zhejiang University, Hangzhou 310003, China

**Keywords:** long noncoding RNA, MALAT1, optical probes, tumour imaging, antisense

## Abstract

With the advent of next-generation sequencing technology, there is rapidly increasing interest in long noncoding RNAs (lncRNAs). The objectives of this study were to develop a novel lncRNA MALAT1 near-infrared optical probe, to evaluate the characteristics of this optical imaging probe *in vitro* and to determine whether it can be used for imaging MALAT1 expression in malignant tumours *in vivo*. Conjugation of Cy5.5 to MALAT1 ASO was accomplished using standard NHS (N-hydroxysuccinimide) ester procedures, and the labelled MALAT1 ASO was purified with a Glen-Pak DNA Purification Cartridge and reversed-phase high performance liquid chromatography (HPLC). The *in vitro* cellular uptake results showed that the percentage of cell binding increased with an increasing final concentration and increased with increasing incubation time for the MHCC-LM3 tumour cell flow cytometry analyses. *in vivo* optical imaging exhibited 5’ (Cy5.5)-MALAT1 ASO uptake in the tumour with a maximum at 30 min p.i. that slowly washed out over time. High contrast to normal tissue was gradually observed from 4 h to 48 h p.i. Tumour-to-normal ratios of fluorescence intensities were plotted as a function of time. The *in vivo* competition assay showed little uptake of the probe into the tumours at any time point, indicating effective competition, selectivity of probe binding and retention by tumours *in vivo*. Our proposed Cy5.5 labelling of MALAT1 ASO can serve as a potent optical probe for *in vivo* imaging of tumour expressing MALAT1. Importantly, the successful development of optical probes provides a basis for specific molecular diagnoses in the field of lncRNAs.

## INTRODUCTION

With the advent of next-generation sequencing technology, there is rapidly increasing interest in long noncoding RNAs (lncRNAs). Despite the outdated opinion that lncRNAs may be mere transcriptional noise, evidence suggests that lncRNAs play a vital role in regulating gene expression, cell metabolism, tumour development and progression [1]. The long noncoding RNA Malat1 (Metastasis-Associated Lung Adenocarcinoma Transcript 1), also known as MALAT-1 or nuclear-enriched abundant transcript 2 (NEAT2), is one of the few biologically well-studied lncRNAs, located on chromosome 11q13, and it was originally found to be overexpressed in early-stage non-small cell lung cancer (NSCLC) [2]. Recently, MALAT1 was found to be overexpressed in many human carcinomas, including hepatocellular carcinomas, pancreatic cancer, bladder cancer, breast cancer, colorectal cancer, gastric cancer, and osteosarcoma [3, 4], suggesting that MALAT1 dysregulation is implicated in the development of many types of cancers [5]. Inhibition of MALAT1 expression may suppress cell proliferation, migration, invasion, and the epithelial-mesenchymal transition, and it may induce cell apoptosis and G2/M cell cycle arrest *in vitro* [6]. These associations suggest that targeting MALAT1 may have important clinical implications because it selectively affects cancer cells or residual cancer cells. Several studies demonstrated that MALAT1 may be a promising target for therapeutic potential *in vivo* [7]. For example, Gutschner et al. [8] reported that injection of antisense oligonucleotide (ASO) into subcutaneous tumours of nude mice can effectively inhibit MALAT1 expression *in vivo* and block the metastasis of lung cancer cells. Based on these studies, we hypothesised that MALAT1 is a potential target for a specific molecular diagnosis.

Functional and non-invasive molecular imaging techniques, such as optical imaging, nuclear imaging, and magnetic resonance imaging (MRI), are gradually being incorporated into every aspect of cancer management. Of these, *in vivo* optical imaging has advantages of not requiring ionising radiation, low cost, and easily generated optical imaging probes [9]. Additionally, optical molecular imaging techniques have become essential tools for providing unique insights into disease pathogenesis, drug development, effects of therapy, and the pharmacokinetic behaviour of drug candidates. In this way, optical imaging is making a substantial impact on basic and translation medical research [10, 11]. Therefore, the development of optical imaging molecules that are selective for tumours for *in vivo* studies is becoming an important field for cancer research [12].

The purpose of the study was to explore the feasibility of development of molecular probes for lncRNA. This study reports the method for preparing molecular optical imaging probes (antisense probes with a Cy5.5 emitter on its 5′equivalent end) for specific targeting of overexpressing MALAT1 tumours cells. We evaluated the characteristics of this optical imaging probe *in vitro* and determined whether it can be used for the imaging of MALAT1 expression in malignant tumours *in vivo*.

## RESULTS

**Figure 1:**
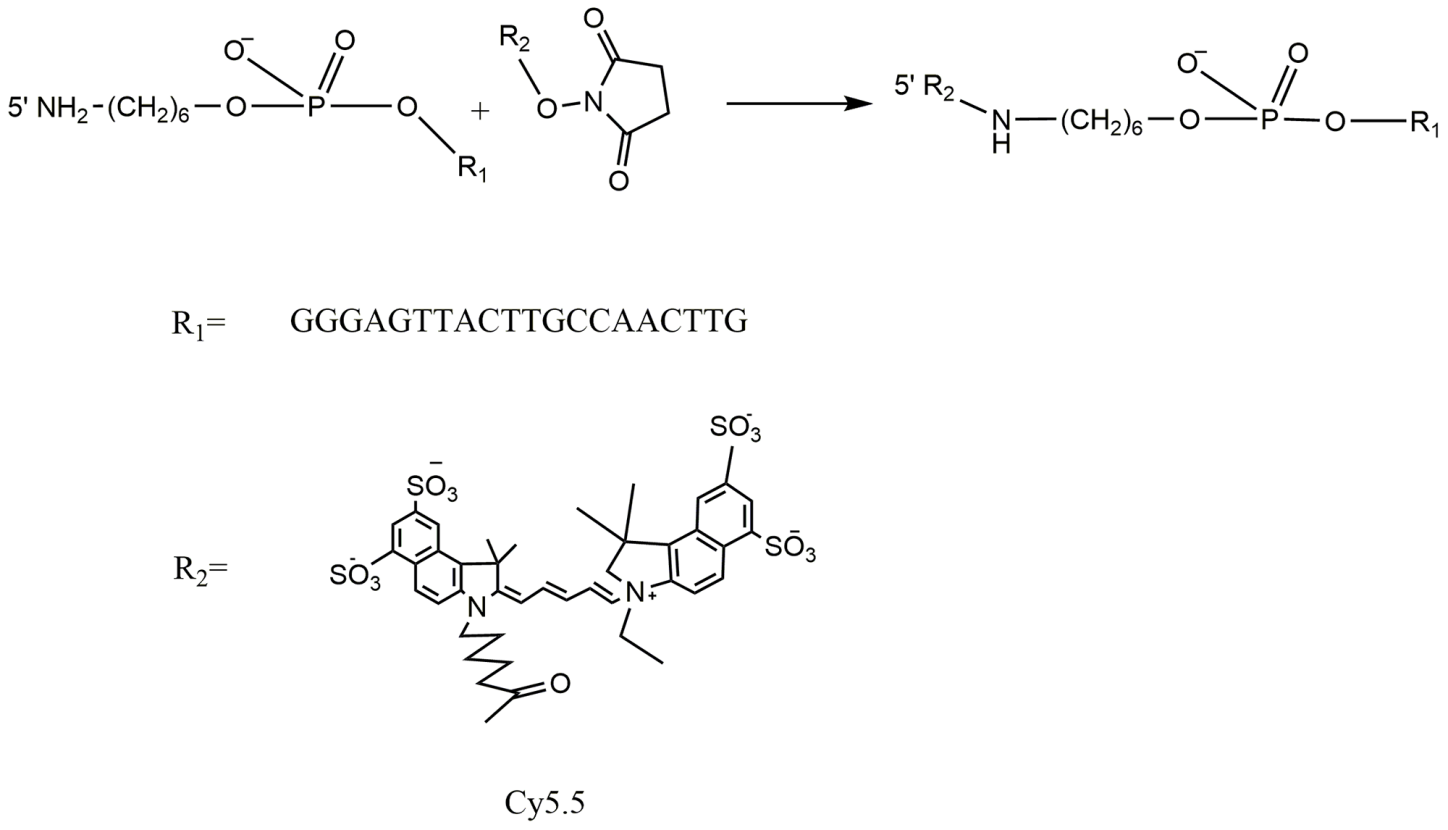
Scheme of Cy5.5 labelled MALAT1 ASO synthesis

**Figure 2:**
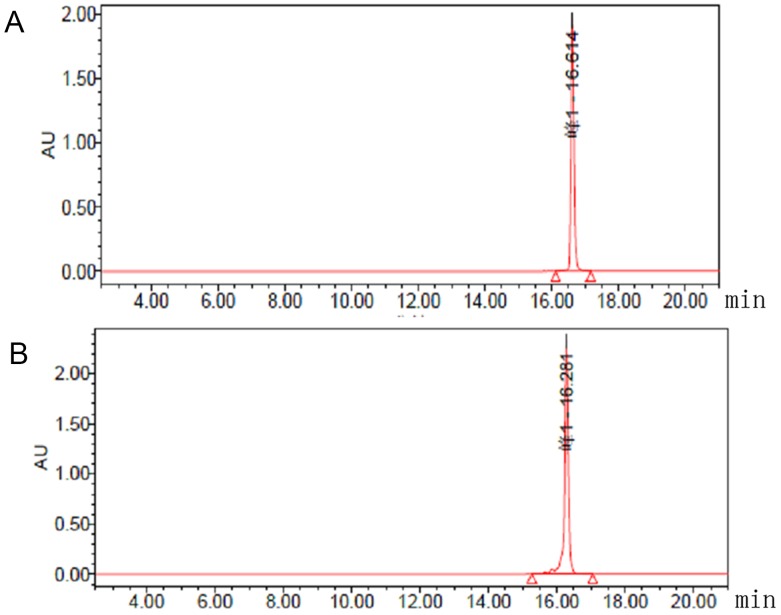
Chromatograms from analytical HPLC of 5’ (Cy5.5)-MALAT1 ASO **(A)** and MALAT1 ASO **(B).**

The schematic molecular structure of the 5’ (Cy5.5)-MALAT1 ASO conjugate is shown in Figure 1. The conjugation of dyes to the ASO was accomplished through standard NHS-ester procedures, and the labelled ASO was purified using a Glen-Pak DNA Purification Cartridge and the ion-pair reversed-phase HPLC method. The isolated fluorescent modified oligonucleotides showed very high purity, nearly 100%, as shown in Figure 2, and were suitable for further study. ESI high-resolution mass spectrometry (M+H)^−^ for 5' (Cy5.5)-MALAT1 ASO (calculated, 7227), and m/z=6149 (M+H)^−^ for MALAT1 ASO. Elution times on the HPLC column were 16.61 min for 5’ (Cy5.5)-MALAT1 ASO, and 16.28 min for MALAT1 ASO.

### *In vitro* cellular uptake results

**Figure 3:**
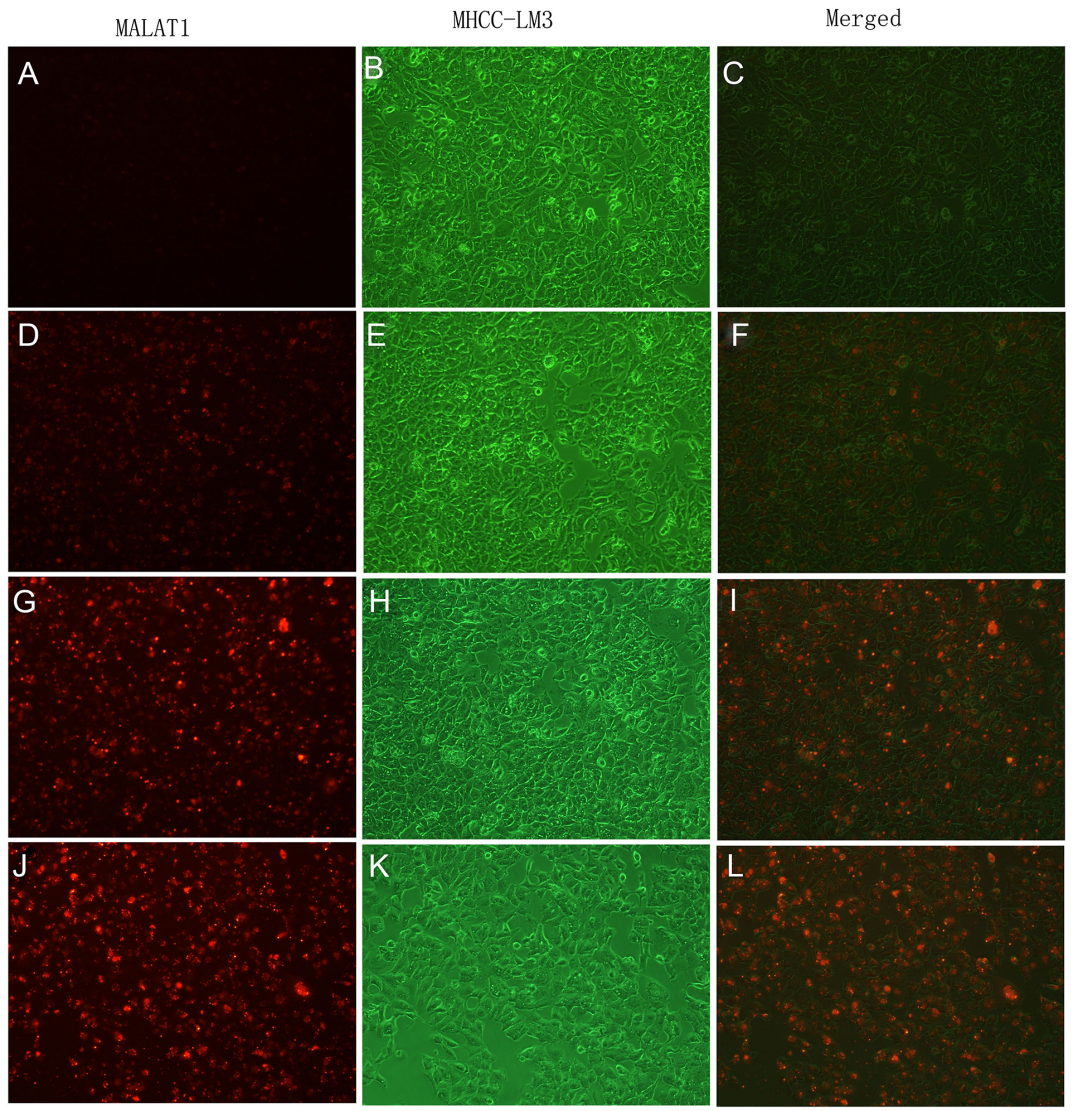
*In vitro* binding of 5’(Cy5.5)-MALAT1 ASO to MHCC-LM3 cells visualised using an Olympus AX70 fluorescence microscope (100×), increasing uptake of 5’(Cy5.5)-MALAT1 ASO by MHCC-LM3 cells with increasing incubation concentrations: 20 nM **(A, B, C),** 50 nM **(D, E, F),** 100 nM **(G, H, I),** and 250 nM **(J, K, L).**

To determine whether the new imaging probes result in specific cellular uptake, uptake studies *in vitro* were performed. The binding specificity of 5’ (Cy5.5)-MALAT1 ASO to tumour cells was determined using fluorescence activated cell sorter (FACS) studies. FACS revealed that the percentage of cell binding (M2) increased with the increasing final concentrations (20 nM, 5.62±0.41%; 50 nM, 30.10±9.78 %; 100 nM, 61.29± 2.32%; 250 nM, 75.57±0.12%). These results suggest that a positive correlation exists between the uptake and final concentration of the 5’ (Cy5.5)-MALAT1 ASO from 20 nM to 250 nM (r=0.91). Figure 3 presents the *in vitro* binding between different concentrations of 5’ (Cy5.5)-MALAT1 ASO and MHCC-LM3 cells observed using a fluorescence microscope.

**Figure 4:**
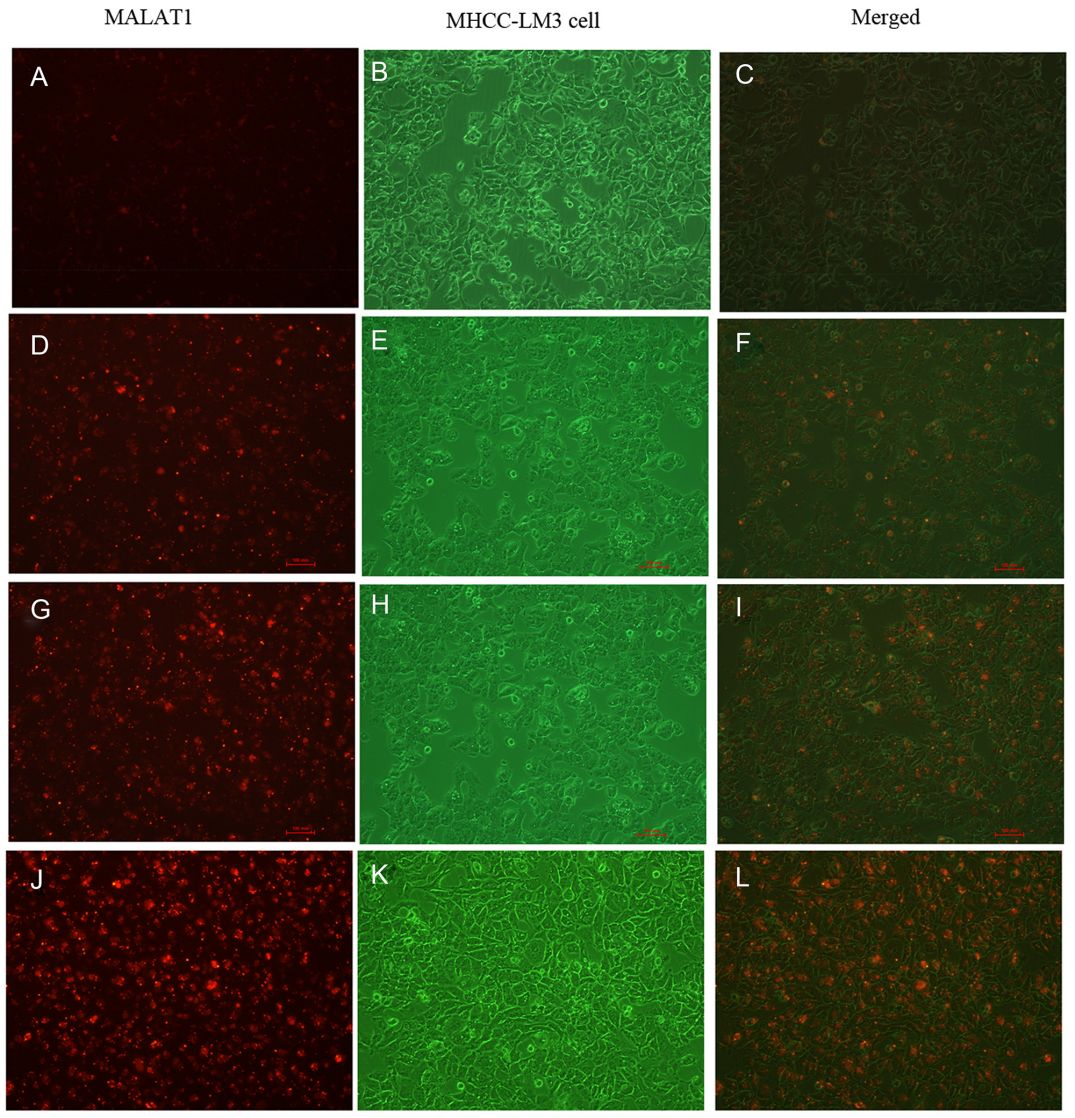
Intracellular uptake of 5’(Cy5.5)-MALAT1 ASO to MHCC-LM3 cells incubated at 37° C at a final concentration of 100 nM with increasing incubation times observed using a fluorescence microscope (100×): 30 min **(A,B,C),** 2 h **(D,E,F),** 4 h **(G,H,I),** 8 h **(J,K,L).**

To investigate the kinetics and intracellular uptake of the 5’ (Cy5.5)-MALAT1 ASO, MHCC-LM3 cells were incubated at 37°C with 5’ (Cy5.5)-MALAT1 ASO at a final concentration of 100 nM at different time. FACS revealed that the percentage of cell binding (M2) increased with increasing incubation times (30 min: 3.55±0.88%, 2 h: 15.66±6.04%, 4 h: 44.77±5.35%, 8 h: 75.28±0.76%), and a positive correlation existed between the uptake and increasing incubation time from 30 min to 8 h (r=0.97). *in vitro* binding of 5’ (Cy5.5)-MALAT1 ASO to MHCC-LM3 cells at different times is shown in Figure 4 under fluorescence microscope.

**Figure 5:**
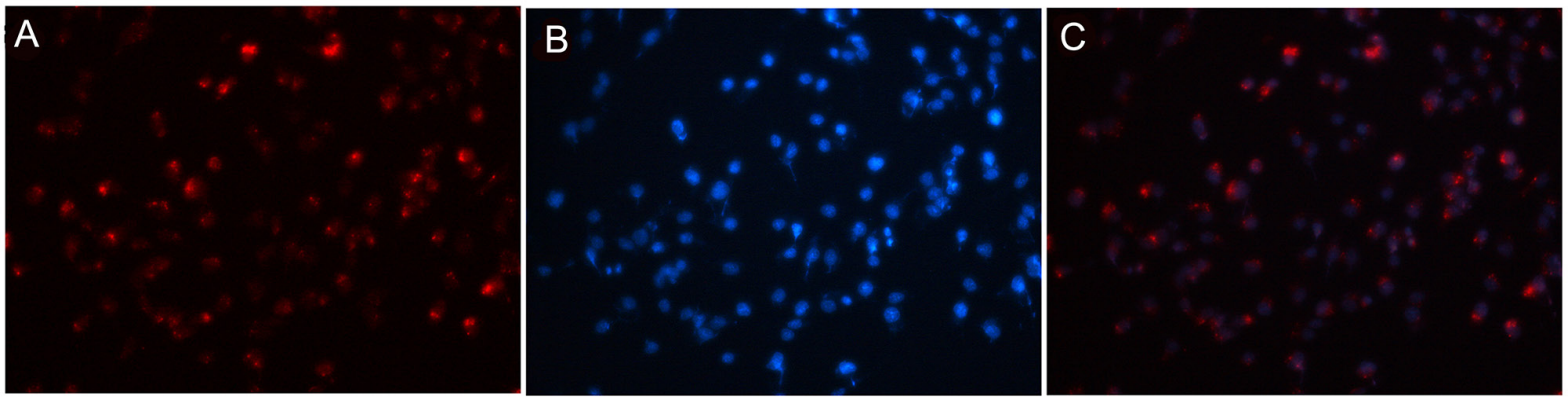
5’(Cy5.5)-MALAT1 ASO is specific and selective for tumour cells overexpressing MALAT1 observed using fluorescence microscopy **(A).** Nuclei were counterstained with DAPI (blue) **(B),** and the accumulation of MALAT1 ASO is retained in the nucleus (merged) **(C).**

The *in vitro* cellular distribution study showed that 5’ (Cy5.5)-MALAT1 ASO is specific and selective for MHCC-LM3 cells using a fluorescence microscope, and the accumulation of 5’ (Cy5.5)-MALAT1 ASO is retained in the nucleus (Figure 5).

### *In vivo* fluorescence imaging

**Figure 6:**
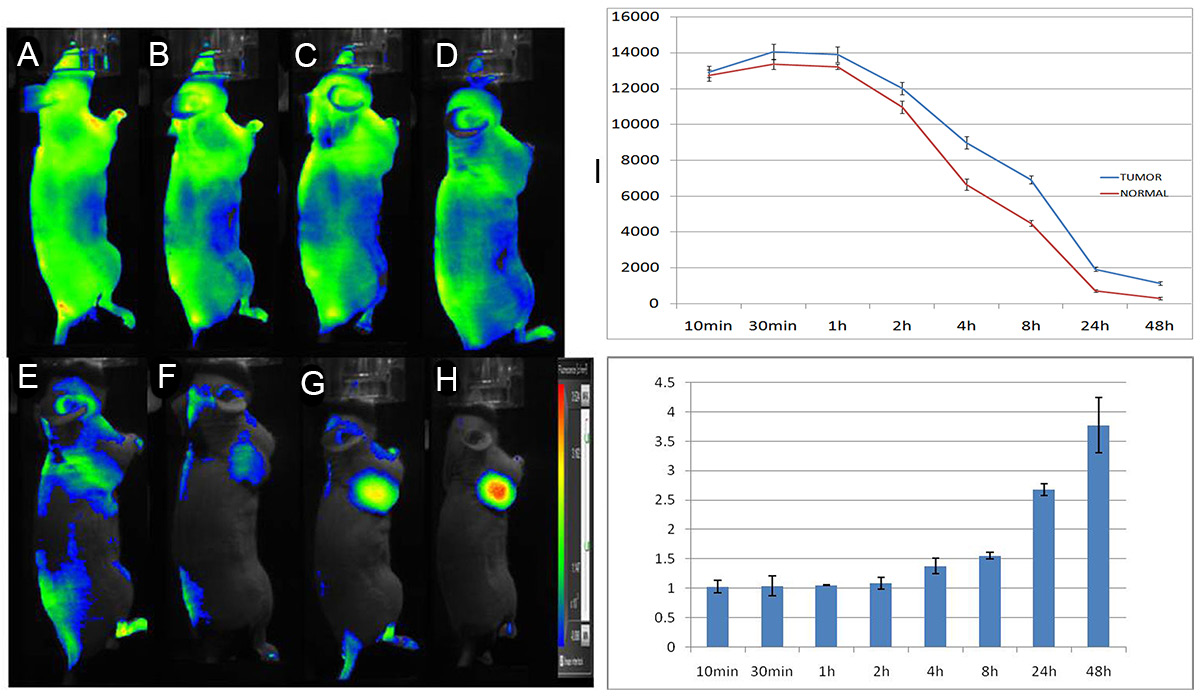
*In vivo* dynamic imaging study of the targeting specificity of 5’ (Cy5.5)-MALAT1 ASO to MHCC-LM3 cell in the right axilla in xenograft mice at 10 min **(A),** 30 min **(B),** 1 h **(C),** 2 h **(D),** 4 h **(E),** 8 h **(F),** 24 h **(G),** and 48 h **(H)** after intravenous administration. Quantification and kinetics of the *in vivo* targeting characteristics of 5’(Cy5.5)-MALAT1 ASO **(I)**. C. Tumour/non-tumour ratios at 10 min, 30 min, 1 h, 2 h, 4 h, 8 h, 24 h, and 48 h **(J)**.

Fluorescence images of tumour-bearing mice administered 5’ (Cy5.5)-MALAT1 ASO probes are shown in Figure 6. 5’ (Cy5.5)-MALAT1 ASO uptake in the tumour was maximum at 30 min p.i. and slowly washed out over time. High contrast to background tissue was gradually observed from 4 h to 48 h p.i., whereas normal tissue had faster probe binding and washout. Tumour-to-normal ratios of fluorescence intensities were plotted as a function of time, and were 1.03±0.08, 1.04±0.07, 1.05±0.22, 1.08±0.03, 1.38±0.13, 1.55±0.04, 2.67±0.10, and 3.77±0.47 at 10 min, 30 min, 1 h, 2 h, 4 h, 8 h, 24 h, and 48 h, respectively (Figure 6J). At early time points, the signals from mouse urine were strong but not retained and became minimal at later time points, suggesting that the probe is mainly excreted from the urinary tract.

**Figure 7:**
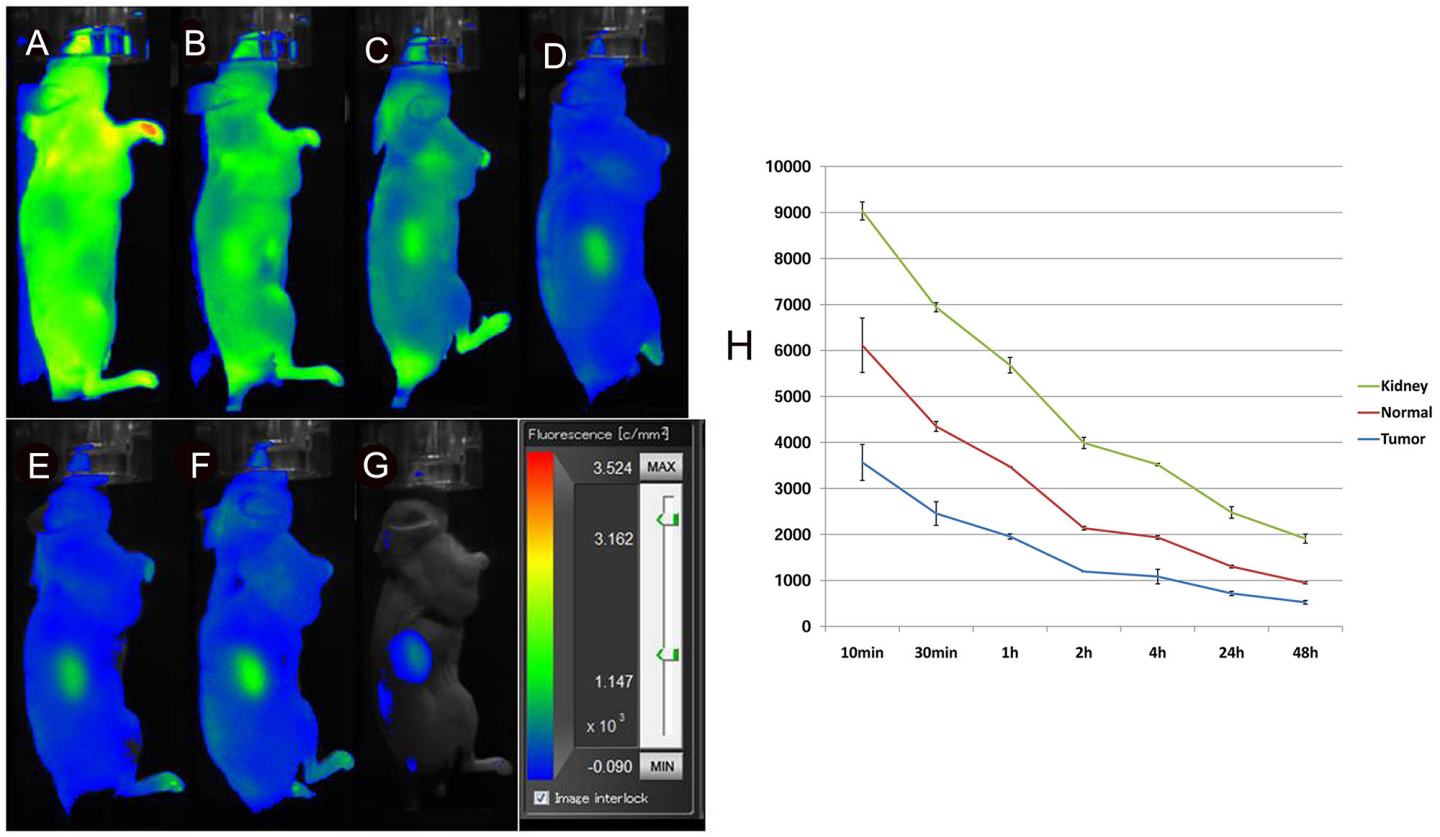
*In vivo* fluorescence imaging of subcutaneous MHCC-LM3 tumour-bearing nude mice in the blocked group The tumour showed little uptake of the probe at any time point up to 48 h after intravenous administration **(A-G)**. Quantification and kinetics of *in vivo* targeting characteristics of 5’(Cy5.5)-MALAT1 ASO **(H)**.

To determine whether the *in vivo* selectivity of probe binding is retained in the tumours, we also performed an *in vivo* competition assay. These studies showed little uptake of the probe in the tumours at any time points, indicating effective competition and selectivity of probe binding and retention by the tumours *in vivo* (Figure 7). Interestingly, signals from the mouse kidney were increased after administration and slowly washed out over time.

**Figure 8:**
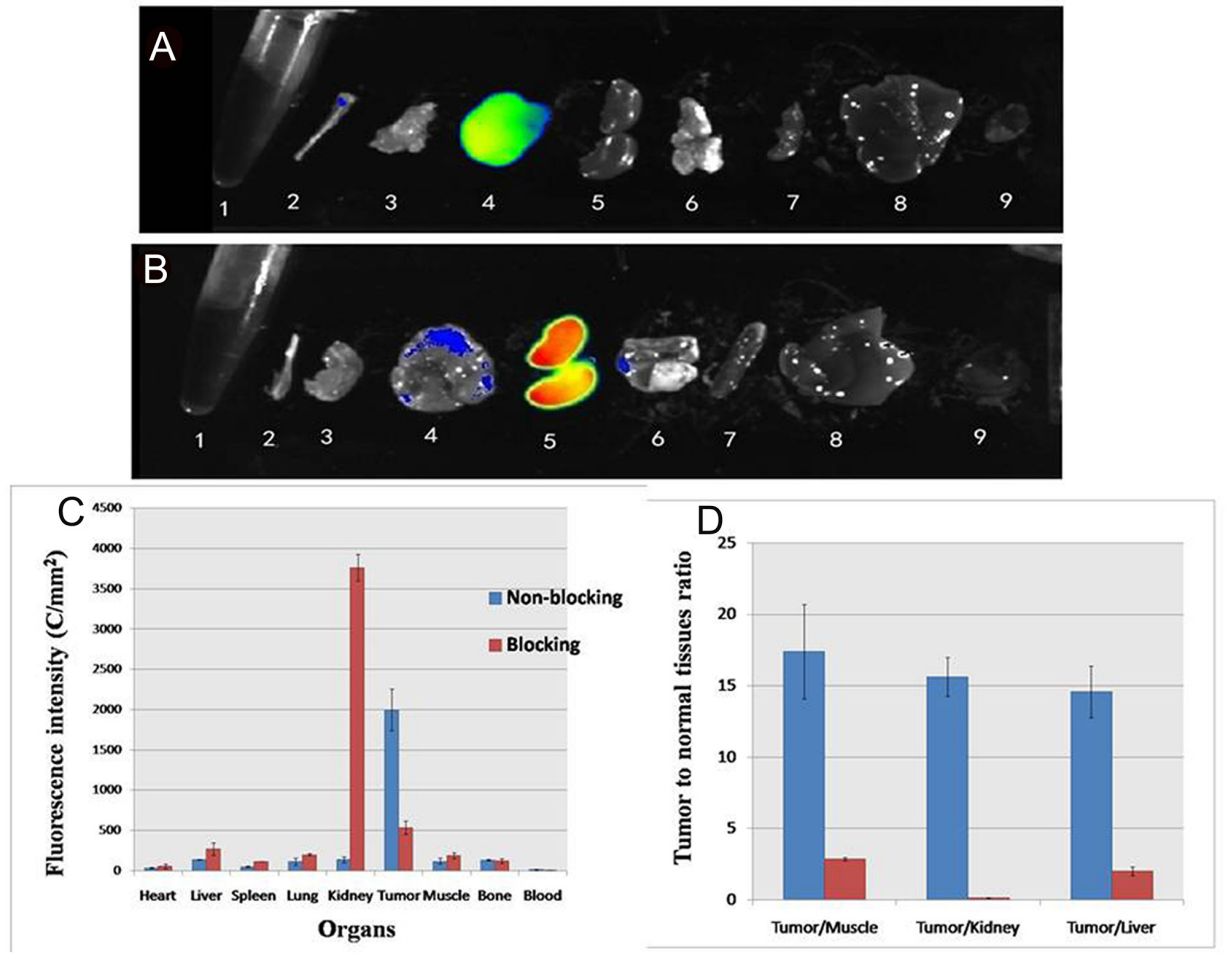
*Ex vivo* imaging of tumor and normal tissues in non-blocking **(A)** and blocking mice **(B)** after euthanizing the mice at 24 h p. i.; 1 blood, 2 bone 3 muscle 4 tumor 5 kidney, 6 lung, 7 spleen, 8 liver, 9 heart. ROI analysis of fluorescence intensity in ex vivo of major tissues with (blocking) and without (non-blocking) co-injection of MALAT1 ASO at 24 hours **(C)**. Fluorescence intensity ratio of tumour-to normal tissue based on the ROI analysis **(D)**. The error bar is calculated as the standard deviation.

Furthermore, excised organs were evaluated ex vivo and quantified using ROI that encompassed the entire organ. The 5’ (Cy5.5)-MALAT1 ASO was predominantly taken up by the MHCC-LM3 tumour at 24 h p.i (Figure 8), as seen in the imaging results *in vivo*. In the blocked group, the unlabelled MALAT1 ASO reduced the overall 5’(Cy5.5)-MALAT1 ASO probe uptake, which is significantly lower in the tumour (p=0.002), suggesting the targeted specificity of the 5’(Cy5.5)-MALAT1 ASO probe. The kidneys showed increased uptake of the 5’ (Cy5.5)-MALAT1 ASO probe, which was identical to the imaging *in vivo*. The contrast ratios of tumour to normal organs for non-blocked and blocked groups were calculated and presented in Figure 8C. Comparison of data between groups demonstrated that 5’ (Cy5.5)-MALAT1 ASO has a tumour-to-muscle ratio of 17.39 ± 2.8) at 24 h p.i. in the non-blocked group and a significantly decreased ratio (2.88 ± 0.11) in the blocked group (Figure 8D). In addition, the non-blocked group ratio of tumour-to-kidney and tumour to-liver uptake at 24 h p.i. was calculated as 15.63 ± 1.36 and 14.60 ± 1.82, respectively, whereas the corresponding values for the blocked group were 0.14 ± 0.03 and 2.02 ± 0.30, respectively.

## DISCUSSION

LncRNAs are a group of RNAs that do not encode proteins, and the lengths of these molecules are greater than 200 nucleotides [16]. Recent observations showed that lncRNAs represent a new frontier in the molecular biology of complex organisms as it is increasingly evident that they are involved in the regulation of almost every stage of gene expression, as well as implicated in cancer onset, progression and a variety of disease states [1], showing a promising future for lncRNA studies. MALAT1, a highly conserved nuclear-abundant lncRNA of approximately 8000 nucleotides, received great interest among lncRNAs since its discovery as an independent prognostic biomarker for early stage metastasising NSCLC, and many studies showed the mechanisms of MALAT1 in regulating gene expression, cell proliferation, invasion, and tumour formation in a broad range of human cancers, including pancreas, breast, colon, liver, oesophageal and prostate cancers [17, 18, 19]. For example, our previous study [15, 19] revealed that MALAT1 overexpression is a predictive factor for tumour recurrence following liver transplantation in hepatocellular carcinoma. The depletion of MALAT1 by siRNA in the HepG2 cell line resulted in a reduction of cell viability, mobility and invasiveness, as well as an increase of sensitivity to apoptosis. Yang et al. [20] suggested that MALAT1 expression in colorectal cancer tissues of human patients with lymph node metastasis was higher than those without metastasis, and MALAT1 may promote colorectal cancer development via its target protein AKAP-9. Several studies showed the aberrant expression of MALAT1 in tumour tissues compared with normal tissues and its association with clinical progression in human cancers, suggesting an important role in cancer pathogenesis and progression [5, 21].

MALAT1 is also an independent prognostic marker for poor outcomes and patient survival in several cancer types, including stage I non-small cell lung cancer and hepatocellular carcinoma [22, 23]. Ma et al. [24] reported that the over-expression of MALAT1 in glioma tissues was positively correlated with grade and tumour size, suggesting that MALAT1 may serve as an authentic prognostic biomarker for patients with glioma. Shuai et al. [24] performed a meta-analysis with a total of 2094 patients from 17 studies to assess the effects of MALAT1 on cancer prognosis and lymph nodes, and the results indicated that the upregulation of MALAT1 expression was significantly associated with poor disease-free survival (HR = 2.29, 95 % CI 1.24-3.35), and recurrence-free survival (HR = 2.09, 95 % CI 0.81-3.37).

MALAT1 has many characteristics that make it attractive as a biomarker and therapeutic targets [1, 25, 26]. MALAT1 can be targeted therapeutically by a variety of approaches, including antisense oligonucleotides, RNAi mediated gene silencing, small molecule inhibitors and by gene therapy [7]. Schmidt et al. [27] reported that RNAi-mediated suppression targeting MALAT1 suppressed migration and clonogenic growth in A549 NSCLCs *in vivo*, exhibiting significant anticancer effects. ASO, which is relatively smaller than siRNAs, give a higher rate of delivery into the nucleus where most lncRNAs are confined [26]. Gutschner et al. [28] observed an accumulation of MALAT1 ASO in both tumour and tumour-associated stromal cells and effectively reduced both human and mouse MALAT1 expression compared with control ASO. Tripathi et al. [13] reported that compared to control cells, MALAT1-depleted cells using ASO showed a consistent reduction in the RNA and protein levels of several of the mitotic genes analysed. To the best of our knowledge, up to now no molecular probes of lncRNA have been reported.

Considering the above factors, we hypothesised that MALAT1 is a potential molecular target and that fluorophore-conjugated MALAT1 ASO would generate a novel molecular probe for the optical imaging of tumours. To create the probe, we selected the Cy5.5 dye, which is used as a promising contrast agent for the *in vivo* demarcation of tumours, to conjugate with the MALAT1 ASO [14, 29]. The synthesis method is convenient, high purity is obtained (nearly 100%) using a Glen-Pak DNA purification cartridge and HPLC method, and it is suitable for further study.

The present study identified a near-infrared molecular imaging probe for cells over-expressing MALAT1 that allows for non-invasive specific detection of tumours *in vitro* and *in vivo*. *in vitro* cellular uptake experiments with 5’ (Cy5.5)-MALAT1 ASO showed that it can selectively bind to and be taken up by MHCC-LM3 cells, and this retention is time-dependent. FACS analyses revealed that the percentage of cell binding (M2) increased with increasing incubation times from 30 min to 8 h, with a positive correlation existing between uptake and increasing incubation time (*r*=0.97). By contrast, 5’ (Cy5.5)-MALAT1 ASO bound to MHCC-LM3 cells in a dose-dependent manner, and there was increased uptake of MALAT1 antisense oligonucleotides by MHCC-LM3 cells with increasing concentrations, ranging from 20 nM to 250 nM. In addition, the intracellular distribution of 5’ (Cy5.5)-MALAT1 ASO was also examined, and the results showed that 5’ (Cy5.5)-MALAT1 ASO was specifically retained in the nucleus, which is identical to a previous study [30, 31].

The whole-body fluorescent images of the MHCC-LM3 cell xenograft mouse model presented in Figure 6 provide further evidence to support the hypothesis that the 5’ (Cy5.5)-MALAT1 ASO can target tumours *in vivo*. The 5’ (Cy5.5)-MALAT1 ASO uptake in the tumour showed a maximum at 30 min p.i. and slowly washed out over time. High contrast to background tissue was gradually observed from 4 h to 48 h p.i. The normal tissue demonstrated rapid probe uptake. However, probe washout in the tumour is much slower than in normal tissue, leading to excellent tumour-to-normal tissue contrast at later time points. 5’ (Cy5.5)-MALAT1 ASO exhibited high contrast tumour-to tissue ratios for the imaging of dissected tissues and organs, which were consistent with the *in vivo* imaging findings.

For the development of new molecular probes for *in vivo* tumour imaging, high specificity binding is an important property [32]. To further demonstrate that binding of our 5’ (Cy5.5)-MALAT1 ASO probe is specific for MALAT1 *in vivo*, competition studies were conducted by injecting unlabelled MALAT1 ASO before injecting 5’ (Cy5.5)-MALAT1 ASO. Significantly reduced tumour uptake of 5’ (Cy5.5)-MALAT1 ASO was observed for the blocked group at all times p.i. (*P* < 0.05), indicating that it is a target-specific probe. In the blocked experiments, signals from the mouse kidney were gradually increased, reaching a peak 24 h after administration, and then gradually decreasing, suggesting that the probe is mainly excreted from the urinary tract.

In summary, our results provide evidence that Cy5.5 labelling of MALAT1 ASO is a convenient approach and that these novel optical probes can serve as specific optical probes for the *in vivo* imaging of tumours expressing MALAT1. More importantly, the successful development of optical probes provides for specific molecular diagnoses in the field of lncRNAs, which deserve further study.

## MATERIALS AND METHODS

### General

Single strand 20-base DNA sequences were modified as phosphorothioates (Shanghai Integrated Biotech Solutions Co., Ltd, China), and the base sequence used was as follows: GGGAGTTACTTGCCAACTTG (MALAT1 ASO) [13]. The Cy5.5 NHS ester was purchased from GE Healthcare Bioscience/GE. The Glen-Pak DNA purification cartridge was obtained from Glen Research (Sterling, VA). Triethylammonium acetate solution (2 M, TEAA), pH 7.0, was purchased from Glen Research (Sterling, VA). All other chemicals were from Sigma-Aldrich (St. Louis, MO).

The HPLC system was equipped with a quaternary pump, variable wavelength detector, vacuum degasser, and ChemStation for system control and chromatography analysis. HPLC was performed using a C18 column (Zorbax ODS 4.6 ^*^ 250 mm). For the HPLC linear gradient, the following buffers were used: A, 2% acetonitrile in 0.1 M TEAA, pH 7; B, 50% acetonitrile in 0.1 M TEAA, pH 7. The column was eluted at 1 mL/min at room temperature. Mass spectra were obtained on a Q-Tof premier-UPLC system equipped with an electrospray interface (ESI) (Waters, USA). The product was collected, lyophilised, and stored in the dark at -20°C until use.

### Synthesis of 5’ (Cy5.5)-MALAT1 ASO

Ten nanomoles of the MALAT1 ASO (GGGAGTTACTTGCCAACTTG) bearing 5’-aminoalkyl linkers was dissolved in 15 μL of 0.1 M NaHCO_3_. A volume of 7 μL of 49 mM Cy5.5-NHS in DMSO was added, and the mixture was incubated at room temperature for 12 h in the dark [14]. Then, 40 μL of water was added, and the modified MALAT1 ASO was separated from the excess dye using a Glen-Pak DNA purification cartridge according to the manufacturer’s recommendations. High-performance liquid chromatography (HPLC) (1200 series, Agilent, USA) was used for the isolation and purification of the product. After Glen-Pak DNA purification, the modified MALAT1 ASO was precipitated by standard ethanol-sodium acetate treatment and purified by HPLC as described above. The purified modified MALAT1 ASO was concentrated in a centrifugal vacuum concentrator.

### *In vitro* cellular uptake study

The human MHCC-LM3 cell line, which was selected for its high MALAT1 expression [15], was purchased from the American Type Culture Collection (ATCC, Manassas, VA, USA). It was cultured at 37°C in a humidified atmosphere of 5% CO_2_ in Dulbecco’s modified Eagle’s medium (DMEM) supplemented with 10% foetal calf serum with 100 U/mL penicillin and 100 μg/mL streptomycin.

MHCC-LM3 cells were detached with trypsin and then counted and plated in 6-well flat-bottomed culture plates in DMEM with 10% foetal bovine serum (2 mL/well, 2.5* 10^6^ cells per well) and incubated at 37°C, 50 mL/L CO_2_ for 24 h, until the cell density reached 80%. In one experiment, MHCC-LM3 cells were incubated for 4 h at 37°C with 150 μL medium (with serum) per well containing different final concentrations (20 nM, 50 nM, 100 nM and 250 nM) of 5’(Cy5.5)-MALAT1 ASO using Lipofectamine 2000 (Invitrogen, Carlsbad, CA) in accordance with the manufacturer’s procedure. In another experiment, MHCC-LM3 cells were incubated for different times (30 min, 2 h, 4 h, and 8 h) at 37°C with a total volume of 150 μL and final concentrations of 100 nM 5’(Cy5.5)-MALAT1 ASO. After incubation of MHCC-LM3 cells with 5’ (Cy5.5)-MALAT1 ASO, the percent transfected cells were screened and sorted by FACS. These experiments were repeated three times, and parallel wells were included in each experiment.

In addition, an *in vitro* cellular distribution study was performed. After incubation of MHCC-LM3 cells with 5’ (Cy5.5)-MALAT1 ASO, the cells were washed twice in 0.1% Tween-20 2× SSC at 56°C for 2 min each, followed by one wash in 0.1% Tween-20 2× SSC at room temperature for 2 min. 4’,6- Diamidino-2-phenylindole (DAPI; Abbott Molecular) was used to stain the cell nucleus, and fluorescence images were collected using an Olympus AX70 fluorescence microscope.

### *In Vivo* and *Ex Vivo* optical imaging

All animal work was performed in accordance with and approved by the Zhejiang University Institutional Animal Care and Use Committee guidelines. BALB/c nu/nu mice (female, 20 ± 3 g, 4- to 6-wk-old; Department of Laboratory Animal Science, Zhejiang University) were used in this study. BALB/c nu/nu mice with MHCC-LM3 cell tumours were randomly divided into 3 groups of 5 mice each. The MHCC-LM3 cell xenograft model was generated by subcutaneous injection of 1.0*10^7^ MHCC-LM_3_ cells resuspended in 100 μL of PBS into the right axilla, and the cells were allowed to grow 3–5 weeks until tumours were 200–500 mm^3^ in volume. The mice were maintained using a standard diet, bedding, and environment, with free access to food and drinking water.

*In vivo* fluorescence imaging was performed using a filter set with an excitation of 658 nm and emission of 719 nm on a Clairvivo OPT plus (Shimadzu Co., Kyoto, Japan). Identical illumination settings (fluorescent dye, excitation mode, exposure time, and field of views) were used for acquiring all images. Fluorescence images were acquired at 15 min, 30 min, 1 h, 2 h, 4 h, 8 h, 24 h, and 48 h after injection of 5’ (Cy5.5)-MALAT1 ASO using 1 s exposure time, anesthetised with 2% isoflurane. The non-blocked mice (n=5) received 100 nmol of 5’(Cy5.5)-MALAT1 ASO injected into each mouse via tail vein and were subjected to optical imaging at various time points post-injection. For the blocked group of mice (n=5), unlabelled MALAT1 ASO (100 nmol) was first injected, and 100 nmol 5’ (Cy5.5)-MALAT1 ASO was injected 30 min later via tail vein. Ellipsoid regions of interest (ROI) of equal area were drawn on the tumour and on the contralateral sites of the left axilla, and the fluorescent intensity was quantified (C/mm^2^).

At the end of the *in vivo* imaging experiments, the mouse was sacrificed by cervical dislocation under isoflurane anaesthesia. The tumours and major organs or tissues (heart, liver, spleen, lung, kidney, muscle, bone, and blood) were collected for ex vivo imaging of the biodistribution of the fluorescent probe, and the fluorescence intensity for each sample was reported (C/mm^2^).

### Statistical analysis

Data are presented as the means ± SD (standard deviation) of n independent measurements. Statistical analysis was performed using Student’s *t* test. A *p* value less than 0.05 was considered statistically significant. Analyses were performed using SAS v8.0 (SAS, Cary, NC, USA).
